# Prevalence of CYP2C19 polymorphism in Bogotá, Colombia: The first report of allele *17

**DOI:** 10.1371/journal.pone.0245401

**Published:** 2021-01-27

**Authors:** Azucena Arévalo-Galvis, William A. Otero-Regino, Gloria N. Ovalle-Celis, Eliana R. Rodríguez-Gómez, Alba A. Trespalacios-Rangel

**Affiliations:** 1 Departamento de Microbiología, Grupo de Enfermedades Infecciosas, Laboratorio de Bacteriología Especial, Facultad de Ciencias, Pontificia Universidad Javeriana, Bogotá D.C., Colombia; 2 Facultad de Medicina, Unidad de Gastroenterología, Universidad Nacional de Colombia, Bogotá D.C., Colombia; University of Colorado Denver Skaggs School of Pharmacy and Pharmaceutical Sciences, UNITED STATES

## Abstract

**Introduction:**

Proton pump inhibitors (PPIs) are a group of drugs that are essential for the treatment of acid-related disorders, such as gastroesophageal reflux (GERD), dyspepsia, gastric ulcers and *Helicobacter pylori* (*H*. *pylori*) infection. PPIs such as omeprazole, esomeprazole, pantoprazole and lansoprazole are metabolized by the CYP2C19 enzyme, which is encoded by a polymorphic gene. Four polymorphisms have an impact on the speed of PPI metabolism: CYP2C19*1/*1 (extensive metabolizers), CYP2C19*2/*2 (intermediate metabolizers), CYP2C19*3/*3 (poor metabolizers) and CYP2C19*17/*17 (ultrarapid metabolizers). Extensive and ultrarapid metabolizers inactivate PPIs quickly, which consequently causes low plasma concentrations of PPIs, while intermediate or poor metabolizers have higher plasma concentrations of PPIs and, therefore, PPIs have greater therapeutic efficacy in individuals with these polymorphisms.

**Objective:**

To determine the frequency of genetic polymorphisms of the CPY2C19 enzyme in Bogotá, Colombia.

**Methods:**

This observational study was conducted in Bogotá between 2012 and 2015 and was part of a clinical trial (ID: NCT03650543). It included 239 subjects with dyspepsia, *H*. *pylori* infection, or GERD symptoms. CYP2C19 genotyping was performed on gastric biopsy samples. Polymorphisms *1, *2, and *3 were analyzed by real-time PCR (Roche^®^), and PCR-RFLP was used to determine the presence of polymorphism *17.

**Results:**

The distribution of different types of PPI metabolizers was as follows: extensive (70.7%), ultrarapid (12.9%), intermediate (8.8%) and poor (0.8%).

**Conclusion:**

The population studied consisted mainly of extensive and ultrarapid PPI metabolizers. These findings show that it is necessary to increase PPI doses in this group of subjects or to use PPIs that are not metabolized by CYP2C19 (rabeprazole). This is the first Colombian work to identify ultrarapid metabolizers.

## Introduction

Proton pump inhibitors (PPIs) are pro-drugs that bind to proton transporters to inactivate gastric acid secretion in parietal cells [1]. PPIs are widely used for the treatment of acid-related disorders, such as peptic ulcers, Zollinger-Ellison syndrome [2], functional dyspepsia [3], gastroesophageal reflux disease (GERD) [4], and between *Helicobacter pylori* (*H*. *pylori*) eradication therapies [5]. The efficacy of PPIs depends on their plasma concentrations and their ability to suppress gastric hydrochloric acid (HCl) [6]. The magnitude of gastric HCl suppression depends on factors such as dose, the timing of taking PPIs with respect to meals and the magnitude of their metabolism by isoenzymes of the cytochrome P450 (CYP) enzymatic system in the liver [7]. This system consists of a large superfamily of integral membrane proteins expressed predominately in the endoplasmic reticulum membrane, in mitochondria and on the cell surface [8]. Although cytochrome P450 is located in different mammalian tissues, it is mainly found in the liver, small intestine and kidney [9]. This superfamily comprises 57 CYP genes and 58 pseudogenes arranged into 18 families and 43 subfamilies in humans, the main function of which is to catalyze different oxidation and some reduction reactions of xenobiotics and endogenous substrates. Cytochrome P450 enzymatic activity is involved in the metabolism of different drugs. In the case of PPIs, some of them (omeprazole, esomeprazole, lansoprazole, dexlansoprazole and pantoprazole) are cleared in the liver primarily by CYP2C19 and to a lesser extent by CYP3A4 [10–13]. CYP2C19 is a 490 amino acid protein that is encoded by the CYP2C19 gene [10]. This gene has 21 polymorphisms, but only four (*1, *2, *3, *17) have been shown to influence PPI clearance [14]. Therefore, subjects will have different abilities to metabolize PPIs depending on CYP2C19 genetic polymorphisms. Thus, subjects can be grouped into ultrarapid metabolizers (UMs) (CYP2C19 *17/*17), extensive metabolizers (EMs) (CYP2C19 *1/*1, which is the wild type, CYP2C19 *1/*17), intermediate metabolizers (IMs) (CYP2C19 *1/*2, CYP2C19 *17/*2, CYP2C19 *1/*3) or poor metabolizers (PMs) (CYP2C19 *2/*2, CYP2C19 * 3/*3, CYP2C19 *2/*3) [10, 14]. Poor and intermediate metabolizer subjects will have greater inhibition of gastric acid secretion since they clear PPI slower than extensive and ultrarapid metabolizers, who will have less inhibition of gastric acid production [7, 10, 14]. The clinical influence of CYP2C19 genetic polymorphisms in the treatment of acid-related disorders where PPIs are required has been evidenced by different studies; for example, it has been shown that the treatment of EMs with GERD can fail when standard doses of PPIs are used independent of age [15–18]. In fact, the prevalence of some gastric diseases where PPIs are used is considerable. Around the world and in Colombia, the prevalence of GERD is 13% [19, 20], with the highest prevalence (more than 25%) in Southeast Europe and South Asia and the lowest (below 10%) in Southeast Asia, Canada, and France [20]. Dyspepsia is a highly prevalent gastric disorder that affects 10% to 15% of the general population [21]. Currently, approximately 50% of North American and European people with dyspepsia receive pharmacological treatment for acid suppression; nevertheless, its efficacy is still controversial. In addition, *H*. *pylori* infection is another remarkable gastric disease in which PPIs are crucial and CYP2C19 genetic polymorphisms play a critical role [22]. The prevalence of this infection is approximately 50% worldwide and at least 60% in Colombia [23].

According to the high prevalence of these gastric-acid-related disorders in Colombia and the relevance of the CYP2C19 metabolizer type on PPI efficacy, the aim of this study was to determine the prevalence of CYP2C19 genetic polymorphisms in a Colombian population and specifically screen for the presence of allele *17 associated with ultrarapid metabolizers of PPIs.

## Materials and methods

### Study design, subjects and samples

This study was an observational research study that was performed in Bogotá, Colombia, from September 2012 to August 2015. The present study was part of a clinical trial (ClinicalTrials.gov ID: NCT03650543) in which 355 subjects were enrolled and 133 subjects who met the strict inclusion criteria were finally included [22]. However, since the findings in this previously published clinical trial were shown to be highly relevant, we considered it important to analyze the prevalence of CYP2C19 genetic polymorphisms but in a larger sample since their distribution in a country would have a substantial impact on the future of personalized therapies with drugs metabolized by CYP2C19 treatments, especially for gastric-acid-related disorders. The ethics committees from the Pontificia Universidad Javeriana and Clínica Fundadores approved the study protocol. The study was performed in agreement with Good Clinical Practice guidelines and the ethical principles of the Declaration of Helsinki [24, 25].

This study included subjects between 19 and 70 years old who were referred for functional dyspepsia or peptic ulcers and recommended to have endoscopy and who agreed to participate in the study. The protocol study excluded pregnant women; subjects with concomitant diseases such as diabetes, mental disorders, gastric atrophy or intestinal metaplasia; subjects with previous gastric cancer; and subjects with previous gastric surgery. The subjects were enrolled randomly in the study during gastroenterology consultation after a gastroenterologist explained to them that the information derived from this research could help to select in a better way the PPIs that are used for patients with peptic acid diseases. Written informed consent was obtained from subjects who met the inclusion and exclusion criteria by the endoscopic service.

Since this study was part of a previous clinical trial, all participants underwent initial endoscopy, carried out at the upper endoscopy service clinic. Endoscopy was performed by an expert gastroenterologist with an Exera Olympus CV 145 video endoscope. Endoscopy was performed after six hours of fasting with the standard methodology and with sedation depending on the tolerance of the procedure (on demand) [26]. During the procedure, biopsies of the upper digestive tract were obtained according to established protocols independent of visible pathologies [27, 28], and a gastric body biopsy sample was taken for the molecular analysis of CYP2C19 genetic polymorphisms. This biopsy sample was transported in 500 μL of Brucella broth (Becton Dickenson^®^) plus 20% (v/v) glycerol (Invitrogen^®^) and was kept refrigerated until it was processed. The QIAamp^®^ kit (QIAGEN) was used to obtain DNA from gastric biopsy samples according to the manufacturer’s instructions.

### CYP2C19 genotyping

CYP2C19 genetic polymorphisms were determined by real-time polymerase chain reaction (RT-PCR) and by PCR-RFLP.

Real-time polymerase chain reaction (RT-PCR) using the LightMix^®^ kit for human CYP2C19*2 and CYP2C19*3 (Roche^®^) was used to determine CYP2C19 *1, *2, and *3 polymorphisms. The reaction used two specific primers synthesizing 133 bp and 164 bp fragments for the CYP2C19 gene, corresponding to polymorphisms *2 and *3, respectively. In addition, specific probes labeled with two different fluorochromes at two different wavelengths for each polymorphism were used to identify polymorphisms by reading in channel 530 for allele 2 and in channel 640 for allele 3. PCR was performed according to the manufacturer’s instructions. Each PCR was carried out in a final volume of 20 μL as follows: 9.4 μL of molecular-grade water (Tib Molbiol), 1.6 μL MgCl_2_, 2 μL primers, 2 μL probes, 2 μL Master mix and 5 μL of DNA. PCR was performed in a LightCycler 1.5. Before initial analysis, color compensation in reading channels was performed to guarantee good results, and quality control for each allele (wild type and mutant alleles 2 and 3) was included for every test. Allelic classification was analyzed by differences in melting temperatures (Tm) (curves obtained) in channel 530 for allele 2, with Tm between 48.6°C and 54.4°C, and in channel 640 for allele 3, with Tm between 53.4°C and 60.8°C, according to the manufacturer’s suggestions.

In addition, nested PCR and RFLP were used to determine the CYP2C19 *17 genetic polymorphism. This PCR was standardized according to previous reports by Baldwin et al. 2007 [29]. This nested PCR and RFLP consisted of two PCRs and a final enzymatic digestion from the product of the second PCR. First, PCR was carried out in a final volume of 10 μL as follows: 3.2 μL of molecular grade water (Sigma^®^), 0.4 μL primers (10 μM), 5 μL Master mix (Promega^®^) and 1 μL DNA. The primer pair used in this first PCR was as follows: forward primer 2C19-1 (5´-GCCCTTAGCACCAAATTCTC-3´) and reverse primer 2C19-1 (3-´ATTTAACCCCCTAAAAAAACACG-5´). Primers were synthesized by Invitrogen USA and amplified a 473 bp fragment, corresponding to CYP2C19 allele 1. The cycling conditions were as follows: 1 minute at 95°C followed by 35 cycles of 30 seconds of denaturation at 95°C, 30 seconds of annealing at 52°C and 30 seconds of extension at 72°C and finally 7 minutes of extension at 72°C. The second PCR was performed using 0.5 μL of the first PCR product. It was carried out in a final volume of 30 μL as follows: 13 μL of molecular-grade water (Sigma), 0.75 μL primers (10 μM), 15 μL Master mix (Promega^®^) and 0.5 μL of DNA and using another set of primers synthesized by Invitrogen USA: forward 2C19-2 (5´-AAATTTGTGTCTTCTGTTCTCAATG-3´) and 2C19-2 reverse (3´-AGACCCTGGGAGAACAGGAC-5´), which amplified a 200 bp fragment. The cycling conditions were as follows: 1 minute at 95°C followed by 25 cycles of 30 seconds of denaturation at 95°C, 30 seconds of annealing at 51°C and 30 seconds of extension at 72°C and finally 7 minutes of extension at 72°C. Next, 15 μL of the second PCR reaction product was incubated with 0.8 μL of NsiI restriction enzyme at 37°C for 8 h. Subsequently, the PCR digestion product was revealed on 2% (w/v) agarose gel and stained with Sybr Safe (Invitrogen^®^) to verify the presence of the 116 bp and 143 bp bands, corresponding to the CYP2C19*1 and CYP2C19*17 alleles, respectively [29]. All details of laboratory protocol are deposited in protocols.oi in dx.doi.org/10.17504/protocols.io.bn46mgze.

In addition, to confirm the presence of CYP2C19*17 by nested PCR and RFLP, 18.4% of samples were selected randomly and sequenced (Macrogen, Korea).

### Statistical analysis

Characteristics of the population, the presence of *H*. *pylori* infection, and CYP2C*19 genetic polymorphism frequencies were analyzed using descriptive statistics, employing the SPSS v.24 statistics program. Bioinformatic analysis of sequences for allele CYP2C19*17 was performed with the BLAST-N tool. The alignments were performed with the wild-type reference sequences (GenBank Access: AL583836 and NG_008384.3.18) [30].

## Results

### Population characteristics

The study included 239 participants from September 2012 to August 2015. Participant distribution was as follows: females 70.3% (168/239) and males 29.7% (71/239). The main reasons for medical consultation were acid reflux 45.2% (108/239) and dyspepsia 41% (98/239). The main endoscopic findings were chronic gastritis (100%; 239/239), erosive esophagitis (45.2%; 108/239) and hiatal hernia (13.8%; 33/239), with additional findings less frequently shown in Table 1. None of the participants smoked or drank alcoholic beverages.

**Table 1:** Characteristics of participants.

Characteristics	Study Participants
**General characteristics**	
Sex (male:female)	71 (29.7):168 (70.3)
Age (years)	47.24 ± 12.6
Weight (kg)	66.7 ± 14.1
Height (cm)	161.2 ± 10.4
Body mass index (kg/m^2^)	26.4 ± 18.1
**Reason for medical consultation**	**% (n)**
Reflux	45.2 (108/239)
Dyspepsia	41 (98/239)
Anemia	1.3 (3/239)
Dysphagia	0.8 (2/239)
Weight loss	0.4 (1/239)
Other symptoms	11.3 (27/239)
**Endoscopic findings**	
Chronic gastritis	239 (100)
Nodular gastritis	4 (1.7)
Erosive esophagitis	108 (45.2)
Grade A	102 (42.7)
Grade B	5 (2.1)
Grade D	1 (0.4)
Hiatal hernia	33 (13.8)
Metaplasia	18 (7.5)
Duodenal ulcer	2 (0.8)
Gastric ulcer	2 (0.8)
Esophageal papilloma	2 (0.8)

### Prevalence of CYP2C19 polymorphisms

**Fig 1:**
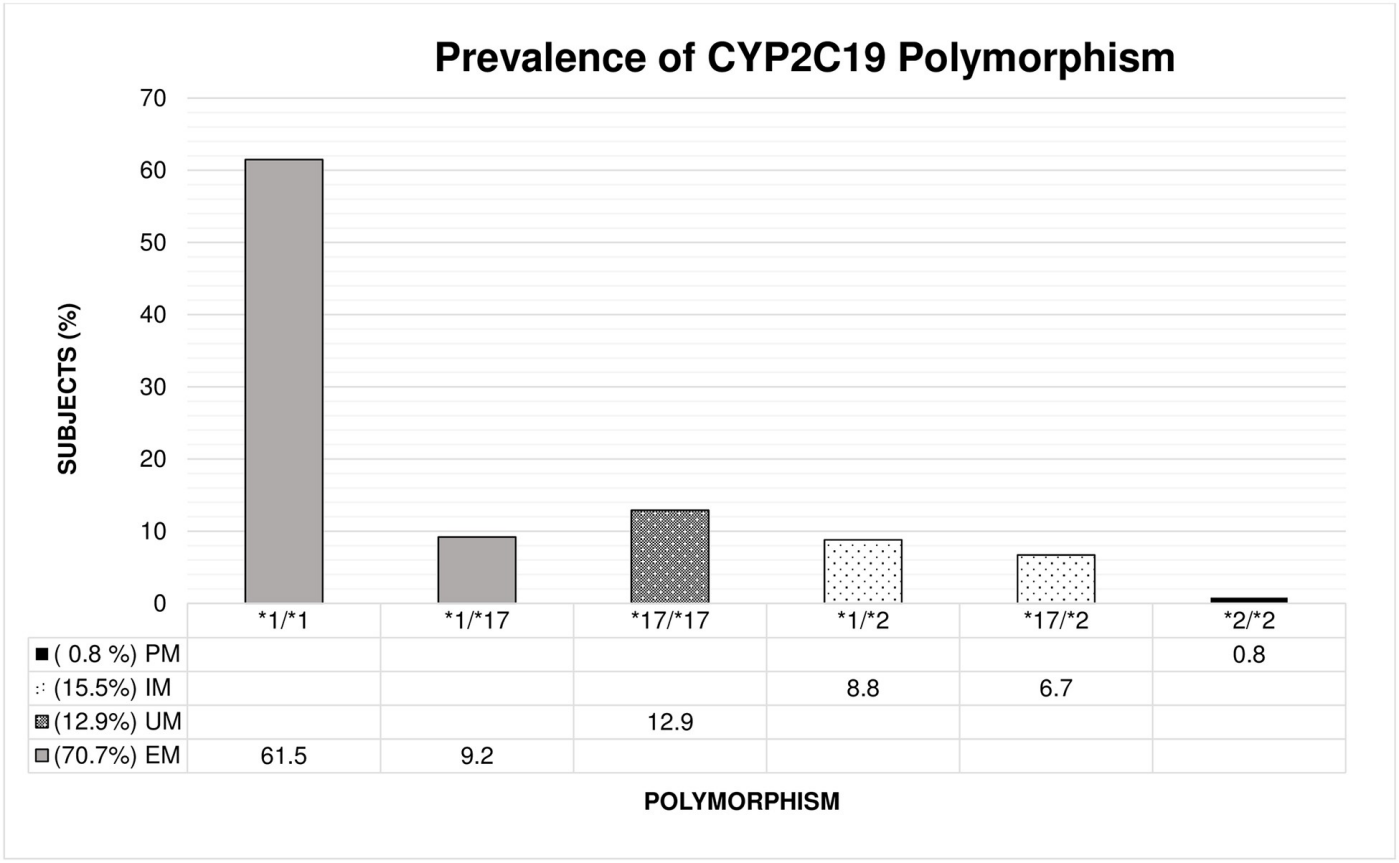
Prevalence of polymorphisms of CYP2C19. The distribution of CYP2C19 polymorphisms in Bogotá, Colombia. The high prevalence of polymorphisms compatible with extensive and ultrarapid metabolizers of PPIs is evident. EM: extensive metabolizer, UM: ultrarapid metabolizer, IM: intermediate metabolizer, PM: poor metabolizer.

The most frequent CYP2C19 genetic polymorphism in the population studied was CYP2C19 *1/*1, found in 61.5% (147/239) of subjects, CYP2C19 *1/*17, which corresponds to extensive metabolizers, was found in 9.2% (22/239) of subjects and CYP2C19 *17/*17, which corresponds to ultrarapid metabolizers, was found in 12.9% (31/239) of subjects. CYP2C19 *3/*3, which corresponds to poor metabolizers, was not present, and the other CYP2C19 genetic polymorphisms analyzed were also present at a low frequency (Fig 1).

**Fig 2:**
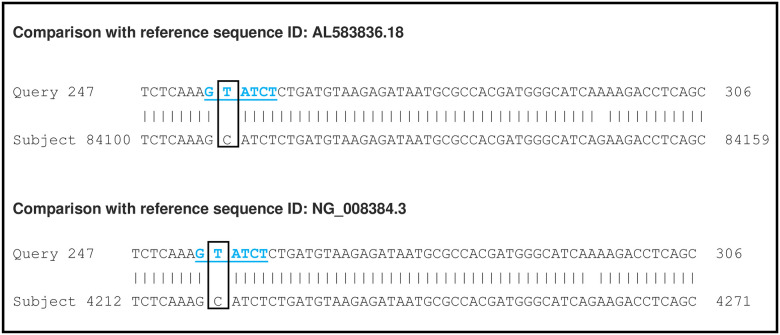
CYP2C19*17 sequence. An example of a CYP2C19*17 sequence analysis. For this purpose, the sequence was compared with two wild-type reference sequences (GenBank Access: AL583836 and NG_008384.3.18). The sequence analysis shows the SNP at position -806(C >T) (quadrate) relative to translation start that is surrounded by the sequence GTATCT (blue underline).

In addition, bioinformatics analysis for the confirmation of CYP2C19*17 showed the genetic single-nucleotide polymorphism (SNP) located at -806 (C > T), which is associated with allele *17 [30] (Fig 2). The sequences are in deposit in NCBI GenBank with the accession numbers: MW261513, MW261514, MW261515, MW261516, MW261517, MW261518, MW261519, MW261520, MW261521, MW261522, MW261523, MW261524, MW261525, MW261526.

## Discussion

This study found that 83.6% (70.7% EMs and 12.9% UMs) of the studied population metabolized the majority of PPIs quickly. These findings are similar to those of a previous Colombian study by Isaza et al. 2007 in another population in this country [31]. However, it is important to highlight that this is the first study in which the presence of an ultrarapid metabolizer was reported to be 12.9%. This prevalence is related to reports in Brazil (15.8%), Sweden and Ethiopia (18%). In Colombia, the prevalence of CYP2C19 *17/*17 is higher than the prevalence of this genotype in African-American (4%) and Asian subjects (< 1%). In contrast, the frequency of poor metabolizers is small (0.85%) in relation to their frequency in Asia (15%), Europe and Africa (2–5%) [11].

Moreover, the frequency of intermediate metabolizers found here was 15.5%; it is small in contrast to that in Asia (45–50%) Europe and Africa (25–35%) [11]. Likewise, the prevalence of extensive metabolizers found in this study (67.78%) was higher than the prevalence reported in African-American (24%) and Asian populations (2–16%) [11]. Thus, these genetic differences make it difficult not only to compare but also to extrapolate treatments between populations around the world. It is important to highlight that although the results of this study may be useful for populations with similar characteristics, it would be ideal to know the prevalence of CYP2C19 genetic polymorphisms in each country or region. This knowledge would allow clinicians to adjust the doses of PPIs and other drugs metabolized by CYP2C19. The high prevalence of extensive (CYP2C19*1/*1 and *1/*17) and ultrarapid (CYP2C19*17/*17) metabolizers found in this study would, in particular, explain the therapeutic failures of *H*. *pylori* eradication when PPIs metabolized (omeprazole, lansoprazole or pantoprazole) by CYP2C19 are used [32]. This situation was evidenced in a recent clinical experiment in our country [22].

GERD is another important disease in which CYP2C19 genotyping is relevant since, in this disease, there are subjects who are refractory to PPIs, which means they have a persistence of symptoms after eight weeks of treatment with double doses of PPIs [26]. They are a heterogeneous group of patients, since they do not have a single cause for refractoriness, and it is still unclear what the therapeutic approach for these subjects should be [17]. Some experts suggest exams such as esophageal physiology (impedance with pH monitoring, esophageal manometry) and upper digestive endoscopy with biopsies of the esophagus. These exams help to rule out functional disorders (functional heartburn and hypersensitivity to reflux), non-acid reflux disease, achalasia and eosinophilic esophagitis [33]. In general, according to some studies, 90% of patients have a functional disorder of the esophagus, such as functional heartburn or hypersensitivity to reflux, even if PPIs are administered correctly [34]. Therefore, some authors have recently suggested that these patients should receive additional empirical treatment with a neuromodulator [35], which can help to control residual secondary symptoms [36]. However, treatment with conventional doses of PPIs was ineffective in another subgroup of patients with this acid-related disorder since they are extensive metabolizers of PPIs, which can affect the success of therapies, as previous studies have demonstrated [16–18]. Researchers have found that refractory GERD is more frequent in extensive metabolizers of PPIs than in intermediate or poor metabolizers, in whom these medications have higher success rates [17, 18]. In agreement with these reports and the findings of the present study, in which 83% of Colombian subjects are extensive and ultrarapid metabolizers, it is necessary to modify treatment for subjects with GERD. For this purpose, a good strategy would be the personalization of therapy according to CYP2C19 genetic polymorphism status before therapy administration. Another option would be to increase doses of PPIs that are metabolized by CYP2C19 according to the prevalence of CYP2C19 genetic polymorphisms in places where its distribution is known. Another possibility would be to use PPIs that are not metabolized by CYP2C19, such as rabeprazole or esomeprazole [11, 37]. The use of the last strategy can avoid the overuse and high economic costs of exams in some patients, such as esophageal functional tests and upper digestive endoscopies with biopsies. In addition, there is an indirect history for this possibility since some authors have found that the symptoms and quality of life of many patients with GERD improve when their medication is changed to esomeprazole [38, 39]. However, there is little information about the effect of the CYP2C19 polymorphism when dexlansoprazole is used [11]. Furthermore, indirect evidence of the relevance of gastric acid is that vonoprazan, a more powerful PPI that acts independently of CYP2C19, improves the symptoms of many patients with GERD that is refractory to conventional PPIs [17, 40].

The impact of CYP2C19 genetic polymorphism on *H*. *pylori* treatment has been demonstrated by different studies, and a recent meta-analysis demonstrated that *H*. *pylori* therapy is less successful in extensive metabolizers of PPIs than in intermediate or poor metabolizers [41]. As an example, in South Korea, it has been documented that extensive metabolizers have a greater risk of therapeutic failure (OR: 1.84 IC: 95% 1.04–2.39) in *H*. *pylori* treatments [42]. This finding has also been demonstrated in other studies [43–45]. However, it is important to highlight that the therapeutic efficacy is not affected when esomeprazole or rabeprazole is used.

The negative effect of ultrarapid and extensive metabolism of PPIs in *H*. *pylori* treatments can be overcome with high doses of PPIs such as 40 mg of omeprazole two or three times a day or equivalent doses if other PPIs are administered [46]. In general, when PPIs are inactivated quickly by CYP2C19, a strong inhibition of HCl results; thus, gastric pH will not rise above 6. This pH level is optimal during *H*. *pylori* therapy, not only increasing *H*. *pylori* replication and allowing the microorganism to be more vulnerable to the action of antibiotics but also allowing improved action of acid-labile antibiotics. Consequently, if there is not good gastric pH control during *H*. *pylori* eradication therapy, the therapy used is less effective [47–49]. Recently, a Colombian study showed that increasing the dose of omeprazole for ultrarapid and extensive metabolizers resulted in a higher eradication rate of *H*. *pylori* [22]. Nevertheless, the CYP2C19 enzyme also metabolizes other medications, such as clopidogrel, phenytoin, imipramine, indomethacin and warfarin [7]; therefore, in patients who receive such medications and do not respond satisfactorily, the results of this study could be taken into account for the corresponding adjustments. Actually, understanding the genetic polymorphisms involved in the metabolism and efficacy of many drugs is important, at least for the treatment of the most prevalent diseases in the world. Since this understanding could improve classic therapeutic schemes, it should be a part of pharmacogenetics applications that focus on precision medicine, especially in personalized therapies [11, 50].

## Conclusion

In Colombia, more than 80% of the population metabolizes PPIs quickly. According to this finding, we suggest in the future to research the optimal doses of PPIs metabolized by *CYP2C19*. As an example, in Colombia omeprazole doses should be adjusted at 40mg twice a day for extensive and ultrarapid metabolizers. The present study opens doors to new pharmacogenetic studies related to other diseases, such as eosinophilic esophagitis [51], hypertension, diabetes or even cancer. Personalized therapy for *H*. *pylori* infection would have a beneficial impact not only for patients but also for health systems around the world.
